# Host M-CSF induced gene expression drives changes in susceptible and resistant mice-derived BMdMs upon *Leishmania major* infection

**DOI:** 10.3389/fimmu.2023.1111072

**Published:** 2023-04-28

**Authors:** Cyrine Bouabid, Sameh Rabhi, Kristina Thedinga, Gal Barel, Hedia Tnani, Imen Rabhi, Alia Benkahla, Ralf Herwig, Lamia Guizani-Tabbane

**Affiliations:** ^1^ Laboratory of Medical Parasitology, Biotechnology and Biomolecules (PMBB), Institut Pasteur de Tunis, Tunis, Tunisia; ^2^ Faculty of Sciences of Tunis, Université de Tunis El Manar, Tunis, Tunisia; ^3^ Department Computational Molecular Biology, Max Planck Institute for Molecular Genetics, Berlin, Germany; ^4^ Laboratory de BioInformatic, BioMathematic and BioStatistic (BIMS), Institut Pasteur de Tunis, Tunis, Tunisia; ^5^ Higher Institute of Biotechnology at Sidi-Thabet (ISBST), Biotechnopole Sidi-Thabet- University of Manouba, Sidi-Thabet, Tunisia

**Keywords:** macrophages, M-CSF, host background, Resistance, susceptibility, transcriptome, *Leishmania*, network propagation

## Abstract

Leishmaniases are a group of diseases with different clinical manifestations. Macrophage-*Leishmania* interactions are central to the course of the infection. The outcome of the disease depends not only on the pathogenicity and virulence of the parasite, but also on the activation state, the genetic background, and the underlying complex interaction networks operative in the host macrophages. Mouse models, with mice strains having contrasting behavior in response to parasite infection, have been very helpful in exploring the mechanisms underlying differences in disease progression. We here analyzed previously generated dynamic transcriptome data obtained from *Leishmania major (L. major)* infected bone marrow derived macrophages (BMdMs) from resistant and susceptible mouse. We first identified differentially expressed genes (DEGs) between the M-CSF differentiated macrophages derived from the two hosts, and found a differential basal transcriptome profile independent of *Leishmania* infection. These host signatures, in which 75% of the genes are directly or indirectly related to the immune system, may account for the differences in the immune response to infection between the two strains. To gain further insights into the underlying biological processes induced by *L. major* infection driven by the M-CSF DEGs, we mapped the time-resolved expression profiles onto a large protein-protein interaction (PPI) network and performed network propagation to identify modules of interacting proteins that agglomerate infection response signals for each strain. This analysis revealed profound differences in the resulting responses networks related to immune signaling and metabolism that were validated by qRT-PCR time series experiments leading to plausible and provable hypotheses for the differences in disease pathophysiology. In summary, we demonstrate that the host’s gene expression background determines to a large degree its response to *L. major* infection, and that the gene expression analysis combined with network propagation is an effective approach to help identifying dynamically altered mouse strain-specific networks that hold mechanistic information about these contrasting responses to infection.

## Introduction

Leishmaniasis, vector-borne diseases, are endemic in various countries of the South. These diseases are caused by protozoan parasites with different clinical manifestations. In mammalian hosts, *Leishmania* are intracellular parasites and the macrophage-*Leishmania* interaction is critical for the outcome of the infection as macrophages are the main host cells for parasite replication. Parasite pathogenicity and virulence are not the only relevant factors that determine the broad spectrum of disease outcomes. The genetic background, which defines the host immune response, certainly contributes to the diversity of disease phenotypes and clinical manifestations. Studies using animal models such as susceptible BALB/c and resistant C57BL/6 mice have definitely helped better understanding the biological relevance of the host genetic profile in the course of *Leishmania* infection. Using gene expression data, we have previously investigated these two mouse models to gain a deeper understanding of the macrophage-*Leishmania* interaction that may influence the fate of the infection (1).

To elucidate mechanistic insights from whole-genome data, recent attempts have incorporated network propagation (2). In this framework, whole genome data is mapped onto networks of interacting proteins and the data is subsequently propagated through the network using stochastic processes such as random walk with restart. This leads to the identification of subnetworks that agglomerate much of the observed signals and identifies structures that may be related to biological function and pathways. Network propagation has been applied in the context of cancer mutations ( (3), multi-omics responses to drugs (4) as well as responses to infections (5).

Previous studies have compared the differential responses of macrophages from resistant and susceptible mice induced by *Leishmania* (6, 7). However, to the best of our knowledge, no study has focused on the impact of the basal transcriptomic profile of macrophages, from different host backgrounds, on their response to *Leishmania* infection.

In this study, we used time-resolved expression data combined with network propagation to investigate how differences in host genetic background could determine the outcome of pathogen infection. We therefore first compared the Macrophage Colony-Stimulating Factor (M-CSF)-induced transcriptomic profiles in uninfected monocytes derived from two different mouse strains with contrasting behavior in response to parasite infection and identified DEGs that reveal profound differences in immune-related transcriptomic landscapes. Next, we studied the temporal responses of the two mouse strains after infection with promastigotes of the protozoan parasite *L. major* and found candidate immune response genes with elevated expression response in the resistant mouse strain compared to the susceptible strain. Using the DEGs from the untreated host transcriptome profiles as seeds, we then performed network propagation with the NetCore method (8), mapped the dynamic infection response signals *via* homology to a large experimentally validated human protein-protein interaction network integrated from various public resources and identified rather distinct, host-driven network modules. Key genes of these modules were validated with time-resolved qRT-PCR experiments in the two mouse strains. In particular, we found large differences in immune response genes and metabolic processes such as oxidative phosphorylation, lipids and arginine metabolism that highlight the variability in host responses to infection.

In summary, we demonstrate that the host’s gene expression background determines to a large degree its response to *L. major* infection, and that the gene expression analysis combined with network propagation is an effective approach to help identifying dynamically altered mouse strain-specific networks that hold mechanistic information about these contrasting responses to infection.

## Materials and methods

### Expression data retrieval, data normalization and experimental design

Microarray data from GSE31997 were used and CEL files were downloaded from the Gene Expression Omnibus (GEO) database (accession number GSE31995 and GSE31996) in R using the GEOquery package (9). These are expression data from BALB/c and C57BL/6 mouse bone marrow-derived macrophages, differentiated using M-CSF (Peprotech) and either left uninfected or infected with the *L. major* live (P) or killed parasite (KP). Gene expression was monitored at five time points (1h, 3h, 6h, 12h and 24h) post infection, with time matched controls.

Data normalization and background correction of the expression values were performed on the raw data using the rma function (10). A matrix describing the experimental design was constructed. In this matrix (called design matrix), we placed the arrays in rows and experiment descriptors in columns.

### Affymetrix mouse gene annotation

The list of Affymetrix identifiers (affy_mogene_1_0_st_v1) was linked to ENSEMBL gene identifiers (Release 101 from August 2022) using Biomart. To this end, of the 53295 Affymetrix probes contained in affy_mogene_1_0_st_v1, 47876 were assigned to ENSEMBL mouse gene identifiers.

### LIMMA analysis

LIMMA analysis was performed on normalized data. The eBayes function of the Limma package was used to detect DEGs between uninfected BALB/c macrophages and uninfected C57BL/6 macrophages (11). Overexpressed genes were considered significant when log2(FC) ≥1 and adjusted FDR P-value ≤0.05. Underexpressed genes were considered significant when log2(FC) ≤-1 and FDR P-value ≤0.05.

### MasigPro analysis

MaSigPro (12) was applied to the rma-normalized temporal data. Treatment and control profiles across the five different time points were compared for each gene and response genes were identified that showed significant temporal expression changes judged with a two-step polynomial regression model. We used a polynomial regression model of degree 2 to identify genes with significant expression differences (FDR<0.1) of the treatment and control dynamic profiles. The “degree” parameter of the “make.design.matrix” function was set to 2 to define the design matrix of the regression model.

### Interaction network and node scoring

As an underlying network scaffold for network propagation we used the human protein-protein interaction network from the ConsensusPathDB database (13). This network has been integrated from 19 different public resources and an interaction confidence value (range: [0,1]) based on several topological- and annotation-based criteria was assigned to each interaction. For analysis we only used interactions with high confidence >0.95. This resulted in a protein-protein interaction network consisting of 10,707 proteins and 114,516 interactions.

Human-mouse orthology was inferred and each node that had a mouse homologue was initially weighted according to its information content with respect to the time-sensitive infection responses. To this end we calculated scores that reflect the dynamic changes of the genes after infection from the MaSigPro analysis similar as in Selevsek et al. (4): For each gene i, the fit of the quadratic regression model is described by the estimated regression parameters and the corresponding p-values for the constant factor, pi0, the linear factor, pi1, and the quadratic factor, pi2, describing the significance of the deviation from the control time course. The score for the gene i is then computed as


$$S_{i}=-\sum_{j}log_{10}\left(p_{ij}\right)$$

if the overall fit was significant and set to zero elsewhere.

### Network propagation

Network propagation was done with the NetCore method (8). This is a semi-supervised method which implements a random walk with restart method based on node core to gain a reranking of the initial node weights. Then, the re-ranked node weights were assigned a p-value by comparison to random graphs. In a second step, all significant nodes were connected to seed nodes to identify network modules that represent highly ranked subnetworks. As seed nodes we used 155 human genes that had a homologue to one of the 202 mouse genes differentially expressed between the untreated states of the two mouse strains. Thus, the significant subnetworks derived from the network propagation were centered at genes that were already different in the untreated states and make up the generic host differences of the two mouse strains.

### Over-representation analysis

We used the ConsensusPathDB Version 35 (14) to compute over-representation of given gene lists with respect to molecular pathways and GO terms. Additionally, we used the Ingenuity IPA Tool to infer upstream regulators being over-represented. In both cases over-representation analysis was based on Fisher’s exact test to calculate a p-value for each biological category (p<0.01).

### RNA extraction and quantitative RT-PCR (qRT-PCR)

Total RNA from uninfected and infected macrophages was isolated using the TRIZOL reagent (Sigma) and prepared using the RNeasy mini kit (Qiagen). RNA was quantified and controlled using NanoDrop ND-1000 micro-spectrophotometer and RNA quality and integrity (RNA Integrity Number, RIN.9) was assessed on Agilent RNA Pico LabChips (Agilent Technologies, Palo Alto, CA). Reverse transcriptions were carried out for each sample in 20 µl final reaction volume using 273 ng of total RNA, 200 Units of SuperScript III enzyme (Invitrogen) and 250 ng of random primers following the manufacturer’s guidelines (25°C 10 min, 42°C 50 min, 70°C 15 min). RT with no template was included as negative control. qPCR experiments were performed using EVA Green chemistry on BioMark qPCR apparatus (Fluidigm) according to manufacturer’s instructions. For each cDNA sample, specific targeted amplification (STA) was achieved with a primer pool that targets all selected genes (14-cycle pre-amplification using TaqMan PreAmp Master Mix (Applied Biosystems) and by following the producer’s instructions: Each qPCR was performed with 1/20 STA dilution, in duplicate. Gene expression was normalized to 4 reference genes and to uninfected macrophages (NI). Values are expressed in fold changes (2-Delta Delta Ct Method) compared to NI macrophage cells.

### Statistical analysis of qRT-PCR experiments

The data are expressed as mean and SD (standard deviation). All graphs generated and related statistical analysis were performed using GraphPad Prism. Significance was reached with p values<0.05. p values are shown as * for p< 0.05, ** for p< 0.01, *** for p< 0.001 and **** for p<0.0001.

## Results

### M-CSF induced basal transcriptomic profiles of BALB/c and C57BL/6 macrophages revealed significant differences in the immune response

**Figure 1:**
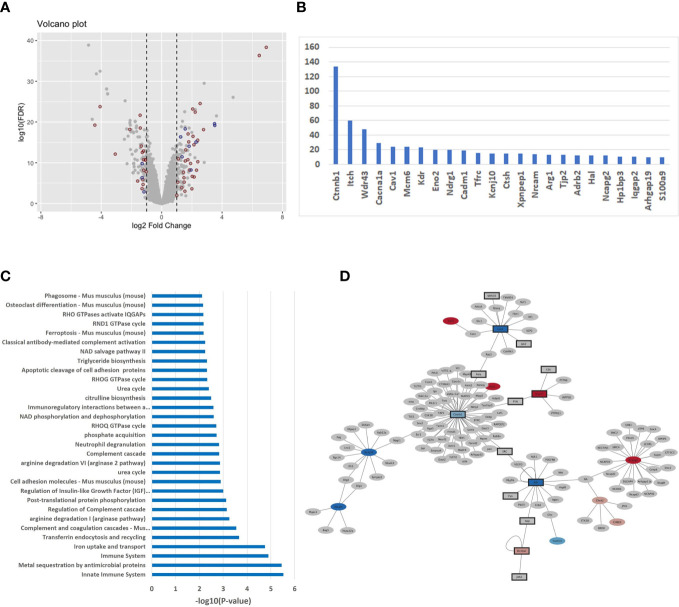
**(A)** Differentially expressed genes (DEGs) in untreated BMdMs reflect consequences for the immune system **(A)** Volcano plot displaying the expression changes of the genes. X-axis: log2 fold change; Y-axis: log10 of the FDR-corrected p-values. Red dots refer to immune response genes (mapped through Affymetrix ID) and blue dots are genes interacting with immune response genes. **(B)** Histogram of DEGs with > 10 mouse protein interactions **(C)** Over-representation of pathways with respect to the DEGs computed with the ConsensusPathDB (version 35). X-axis: -log10 of the pathway enrichment value; Y-axis: Pathway names. **(D)** The PPI network of DEGs extracted from mouse IntAct was constructed using Cytoscape (version 3.9.1).

In order to unravel a potential difference in transcriptomic signatures dependent of the host backgrounds, we compared the M-CSF dependent gene expression profile in non-infected BMdMs derived from BALB/c and C57BL/6 mice. Differentially expressed genes (DEGs) were identified applying a combination of a fold change threshold ≥ 2 and a corrected *p*-value< 0.05. Specifically, 265 probe sets were mapped to 103 mouse genes that are upregulated in differentiated BALB/c BMdMs and 99 mouse genes that are upregulated in differentiated C57BL/6 BMdMs. This result highlights the existence of a differential basal transcriptomic profile between the two host backgrounds (**Figure 1A**).

Over-representation analysis performed with the ConsensusPathDB tool on the 202 DEGs revealed immune-related, pathways and gene ontology (GO) terms, as the dominant difference between the two basal transcriptomic profiles. In total, 49 genes (about 25%) belong to the immune response and enrich pathways of the immune system (**Figure 1C**) such as “innate immune system” (q-value = 3.96E-04), “immune system” (9.47E-04), “complement and coagulation cascades” (1,05E-02) and immune-metabolism pathways such as “iron uptake and transport” (9.93E-04), “arginine degradation” (1.77E-02) and “citrulline biosynthesis” (3.60E-02): Smpdl3b, Ifitm1, Ifitm6, Lpin1, Cxcl9, H2-T24, Apobec3, C1qa, Ada, Trim5, Cadm1, Alpk1, Pianp, Itch, Ifi214, Clec4e, H2-M2, C1qb, Ifi213, Ccrl2, Slamf7, Fcγrt, Ctsh, Lcn2, Klrk1, Trim12a, Ccl8, Trim12c, Ifi44l, Ccr3, S100a9, Ltf, Il-1β, Ifi202b, Cav1, C5ar2, Ifi44, Nlrp1a, Cfh, Nlrp1b, Ifi206, Arg1, Arg2, Ly9, Ifi203, Tfrc, Colec12, Clec2i, Cxcl14. This result is also observable with respect to GO terms with immune system functions as the top categories (**Supplementary File 1**). In order to identify potential regulatory differences of the basal transcriptomic profiles we performed analysis with the IPA system and searched for over-represented upstream regulators with the DEGs (**Supplementary File 1**). One of the top hits is the cytokine IFNγ (p-value = 1.18E-11). Other enriched cytokines include IL4 (3.42E-11), IL6 (3.10E-10), CSF2 (3.37E-09) and TNFα (3.85E-07).

We integrated 12 different mouse interaction databases (Bind, Biogrid, CORUM, DIP, InnateDB, IntAct, MINT, MIPS-MPPI, MatrixDB, PDB, PDZBase and Reactome) with a total number of 11,825 experimentally derived binary interactions and found that about 50% of the DEGs (99 out of 202) have interactions with other mouse genes (**Supplementary File 1**). The number of interactors differs between 1-134. **Figure 1B** shows the genes with 10 or more interactors, among them important immune system genes, for example Itch (60 interactors), Cav1 (15), Cadm1 (16), Tfrc (17) and Ctsh (18).

To further evaluate the strong link between the DEGs and the immune system, we screened IntAct mouse database for immune system genes and their interactors and used Cytoscape (version 3.9.1) to illustrate the results. We found a total of 72 of the remaining genes that are directly connected to immune system genes which underlines the variability of host-driven immune responses in the two mouse strains (**Figure 1D**).

### *Leishmania* infected BMdMs reveals a more robust immune response in C57BL/6 BMdMs

Our results showed variability in the expression of immune system genes between the macrophages differentiated from the two mouse strains. We hypothesized that these differences may have a direct effect on the immune response of the two mouse strains to *Leishmania* infection. We thus compared the transcriptome landscapes in *Leishmania* infected BALB/c and C57BL/6 macrophages and extracted DEGs at different time points after infection (1h, 3h, 6h, 12h and 24h) compared to time-matched uninfected states. The results revealed 789 DEGs for BALB/c and 753 DEGs for C57BL/6 with 488 DEGs in common that were significantly differentially expressed at any of the time points (**Supplementary File 2**).

**Figure 2:**
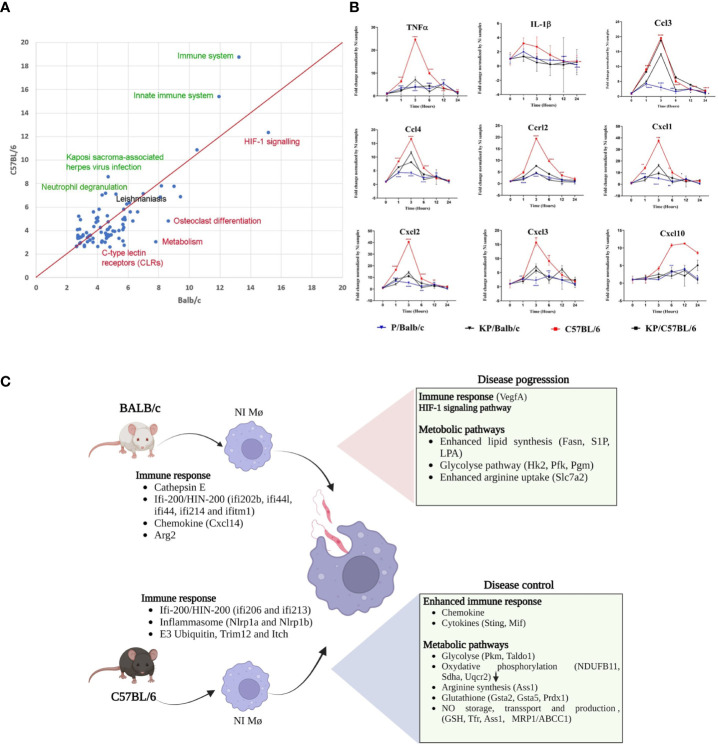
*Leishmania* infected BMdMs reveal an enhanced immune response in C57BL/6 BMdMs. **(A)** Pathways commonly over-expressed by *Leishmania* infected BMdMs in BALB/c and C57BL6 across different time points. X-axis: -log10 of the enrichment q-value in BALB/c; Y-axis: -log10 of the enrichment q-value in C57BL6. Green highlighted pathways show highest difference in enrichment with respect to C57BL6, red highlighted pathways show highest difference in enrichment with respect to BALB/c. “Leishmaniasis” pathway (black) is equally enriched in both mouse strains. Red line: equal enrichment. **(B)** BALB/c (blue filled triangle) and C57BL6 (red filled square) BMdMs were infected with live parasites or killed parasites (black filled triangle and black filled square respectively) for different times. qRT-PCR targeting Cxcl1, Cxcl2, Cxcl3, Cxcl10, Ccl2, Ccl3, Ccr2, IL-1β and TNFα were performed. The graphs show fold change results expressed as the mean ± SD from three independent experiments. *p< 0.05, **p< 0.01, ***p< 0.001, ****p< 0.0001 (two-way ANOVA). **(C)** M-CSF differentiated resistant and susceptible macrophages display different transcriptome profiles emphasizing immune response. The DEGs can modulate the macrophage response during leishmaniasis and explain the enhanced immune response that favor disease control in C57BL/6 macrophages and the M2 polarization of infected BALB/c macrophages. Created by Biorender.com.

To gain insight into the cellular and molecular functions of *Leishmania*-induced host genes, DEGs were subjected to pathway analysis. 95 pathways annotated by multiple resources are enriched by differentially expressed genes after *Leismania* infection in both mouse strains (q<0.01). Comparison of pathway enrichment scores shows that immune, innate immune systems and related pathways are more significantly modulated in C57BL/6 BMdMs whereas metabolic pathways were more enriched in BALB/c BMdMs (**Figure 2A**). Additionally, we found a total of 197 response genes annotated with “Immune response” (GO:0006955). Among these 197 immune response genes modulated by the infection, 41 are unique to BALB/c, 60 unique to C58BL/6 BMdMs and 96 are common to BMdMs derived from both susceptible and resistant mice (**Supplementary File 2**).

To validate the result, we performed qRT-PCR targeting a set of common immune response genes containing Ccl3, Ccl4, Cxcl1, Cxcl2, Cxcl3, Cxcl10, Ccrl2, TNFα and IL-1β. Experiments were performed using the mRNA of BALB/c and C57BL/6 derived macrophages infected with live or heat-killed parasites. As shown in **Figure 2B**, the transcription of this set of parasite-induced immune response genes is 2 to 6 times higher in C57BL/6-derived macrophages. Moreover, compared to the transcription induced by inactivated parasites, the transcription of most of these genes seems to be repressed by live parasites in BALB/c derived macrophages. Thus, we observe that disease control associated with an enhanced immune response and disease progression associated with a weaker immune response and compensatory activation of dedicated metabolic pathways could be driven by M-CSF induced transcriptome profiles that prevail in differentiated resistant and susceptible macrophages (**Figure 2C**).

### Network propagation reveals different host response networks after *Leishmania* infection

In the next step, we were interested in the different biological processes that were affected by the variable host immune responses of uninfected macrophages. For this, we analyzed the time-resolved expression data of infected BMdMs in both mouse strains by a polynomial regression model and defined a score for each gene that reflects the dynamic change of its temporal expression profile (cf Methods) similar to Selevsek et al. (4). We then mapped the mouse genes to human homologs and applied network propagation of the gene scores on a large integrated human protein-protein interaction network using NetCore. Network propagation results in a re-ranking of the input scores for each network node and finally identifies network modules connecting the significantly re-ranked network nodes with the seed nodes that consist of the DEGs derived from the comparison of the uninfected states (cf. Methods). By this approach we derive for each mouse strain its dynamically altered interactome centered at the genes that reflect the differences in the basal host immune profile.

**Figure 3:**
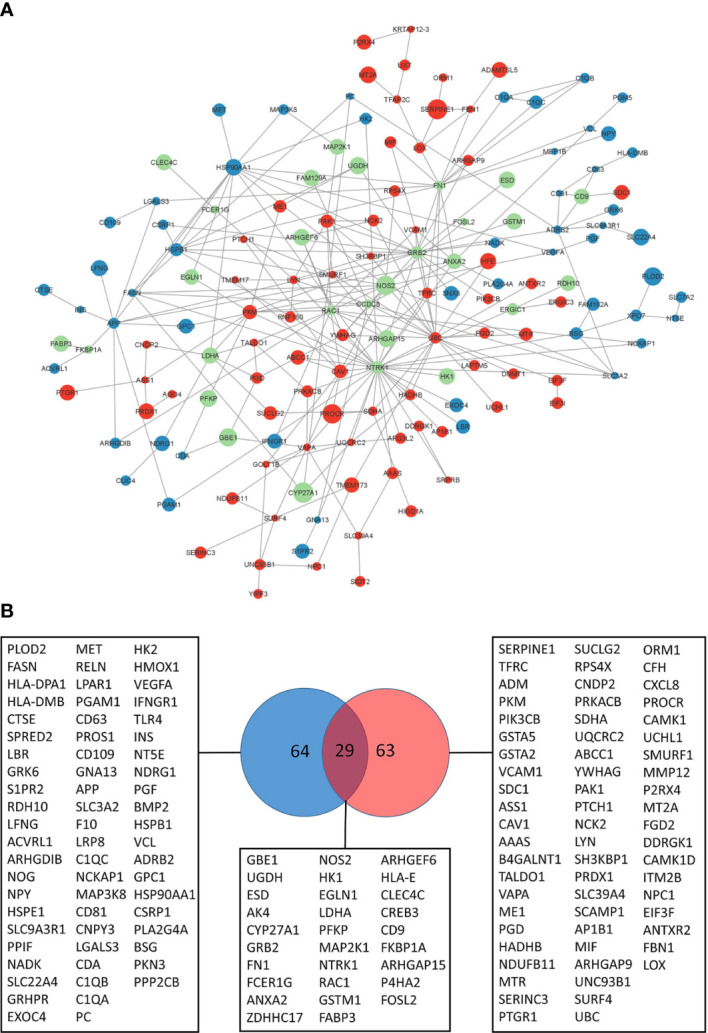
Leishmania response network. **(A)** Largest response network module merged from BALB/c and C57BL/6 network propagation analyses. Color of the nodes reflect to genes identified in the BALB/c response network (blue), C57BL/6 response network (red) or both (green). Size of the nodes reflect the computed network propagation weights. The graph was generated with Cytoscape (version 3.9.1). **(B)** VENN diagram of genes in response pathways (**Supplementary File 3**) enriched for BALB/c (blue) and C57BL/6 (red) network modules genes.

Network propagation yielded subnetworks of 110 and 120 genes dynamically altered by BALB/c and C57BL/6 host responses respectively (**Supplementary File 3**; **Supplementary Figures 1A, B**). The largest components in BALB/c and C57BL/6 are subnetworks of 76 and 95 genes respectively. It can be observed that these modules reflect profound differences of the dynamic host responses with the C57BL/6 network being much more densely connected than the BALB/c network (**Supplementary Figures 1C, D**). We then merged the different BALB/c and C57BL/6 response networks and found that C57BL/6-only genes, and particularly the genes that are in both response networks, are more central to the core of the network with more interactions to each other whereas BALB/c-only response genes are more located at the periphery of the graph with lower numbers of interactions (**Figure 3A**). Indeed, the BALB/c-only nodes had an average node degree of 2.5, the C57BL/6-only genes an average node degree of 3 and the genes common to both had an average node degree of 5 (**Supplementary Figure 2**).

We were interested in the pathway content of the network modules and extracted 156 genes of the BALB/c and C57BL/6 network modules that enrich molecular pathways. Pathway over-representation of the module genes leads to 65% pathways that are different between the two strains (**Supplementary File 3**). Thus, the pathway contents of the computed network modules are fairly distinct and indicate that the differences in basal transcriptome profiles lead to highly different responses to infection. Indeed, only 29 of the 156 genes that enrich pathways are common to both response networks (19%) whereas 64 and 63 genes respectively are unique to the BALB/c and C57BL/6 response networks. This heterogeneity reflects different functional consequences in the host’s responses to the pathogen (**Figure 3B**).

### Network propagation allows retrieval of genes and pathways reported to be affected by *Leishmania*

The propagation centered at the variable host’s immune systems allowed us to recover a significant part of the genes and biological processes previously reported as modulated and altered in response to *Leishmania* infection as well as novel associations. For example, regarding the immune system, in the BALB/c module we found the Vascular endothelial growth factor A (VegfA) that was reported to be expressed in the C57BL/6 mice lesions but not in those of BALB/c (15). In the C57BL/6 network module, we observe the presence of Cxcl8/IL-8 a chemotactic factor that attracts neutrophils, basophils, and T-cells, but not monocytes, Sting that mediates type I interferon immune response and the Macrophage migration inhibitory factor (Mif), a key player in the innate immune response.

We also found different genes coding for enzymes regulating metabolic pathways, including glycolytic enzymes. Their transcription is induced at different levels and with different kinetics in macrophages from the two mice strains. Of these enzymes, Hexokinase 1 (Hk1) and lactate dehydrogenase (Ldh) were shared by the BALB/c and C57BL/6 network modules, whereas Hexokinase 2 (Hk2), Phosphofructokinase (Pfk) the gate controller of glucose flux into glycolysis, and phosphoglucomutase (Pgm) are present in the BALB/c module. Pkm that encodes the pyruvate kinase enzyme, catalyzing the final step in glycolysis, is part of the C57BL/6 module. Using qRT-PCR experiments, we were able to validate different temporal response patterns in these genes with respect to either one or both mouse strains (**Supplementary Figure 3**).

Genes coding for the Hydrogenase Ubiquinone Oxidoreductase (Ndufb11), the Succinate Dehydrogenase Complex Flavoprotein Subunit A (SdhA) and Ubiquinol-Cytochrome C Reductase Core Protein 2 (Uqcrc2), component of the three multisubunit complexes of the mitochondrial respiratory chain and a source of reactive oxygen species (ROS), are present in the C57BL/6 network module.

Unique to BALB/c we found different genes coding for components of the lipid metabolism. These include the transcript of the multifunctional fatty acid synthase enzyme FASN and two bioactive lipid molecule receptors, the sphingosine 1-phosphate (S1P) receptor and the lysophosphatidique acid (LPA) receptor. The response network of C57BL/6 contains the Zinc Finger DHHC-Type Palmitoyl transferase 17 (Zdhhc17).

### Network propagation highlights profound differences in the arginine biosynthesis pathway in response to infection

The network analysis allowed us to highlight differences in the regulation of a given pathway between the BMdMs of different origin. One of these is arginine metabolism. L-arginine is required, through the expression of inducible nitric oxide synthase (iNOS), for the Nitric oxide (NO) generation that plays an important role in host killing mechanisms. This amino acid can also be catabolized by arginase to produce polyamines, crucial for parasite replication (16). As can be seen from **Figure 3A**, NOS2 is a highly connected gene with respect to the identified modules. Our analysis shows that while NOS2 is shared by macrophages derived from both mice strains, argininosuccinate synthetase 1 (Ass1), the rate-limiting enzyme for citrulline recycling into arginine, is unique to the C57BL/6 network module (**Figure 3A**).

**Figure 4:**
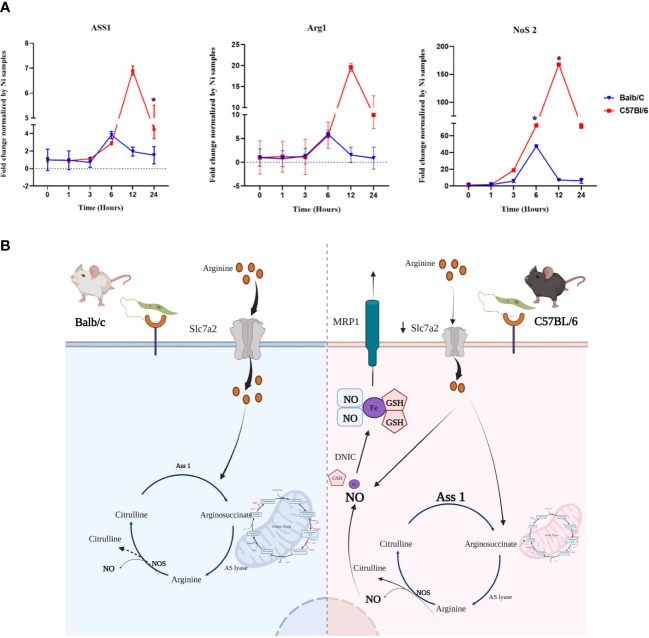
Genes involved in the arginine metabolism is differentially regulated by *Leishmania* in susceptible and resistant bone marrow-derived macrophages macrophages (BMdMs). **(A)** Balb/c (*blue (Parasite (P)) black (Killed P (Kp)) filled triangle*) and C57BL6 (*red (P) black (Kp) filled square)* BMdMs were infected by *L. major* promastigotes for different times. qRT-PCR targeting argininosuccinate synthetase 1 (Ass1), arginase 1 (Arg1) and nitric oxide synthase (Nos2) were performed. The graphs show fold change results expressed as the mean ± SD from three independent experiments. *p<0.05 (two-way ANOVA). **(B)** Hypothetical arginine metabolic regulation in C57BL/6 and BALB/c derived macrophages. The expression of the arginine transporter, SLC7A2, is reduced in C57BL/6 macrophages which decreases their uptake of extracellular arginine. This could be considered by the cell as a depletion of extracellular arginine and drive the activation of Ass1 for citrulline recycling. Arginine from citrulline recycling is the preferred substrate for NO production and therefore iNOS activation. This enhanced production of NO and the accumulation of glutathione (GSH) could result in the generation of dinitrosyl iron complexes (DNIC) that will be exported through the multidrug resistance protein 1 (MRP1). Created by Biorender.com.

qRT-PCR experiments performed on mRNAs from infected BALB/c and C57BL/6 BMdMs, show that transcriptions of Arg1, Nos2 and Ass1 are induced by the parasite in BMdMs of both mouse strains. During the first hours, the kinetic and the level of this transcription were comparable in the macrophages derived from the two mice strains. However, starting from 6h post-infection the transcription of these genes decreased in BALB/c BMdMs while continuing to increase in C57BL/6 BMdMs (**Figure 4**) to reach their maximum 12 h post-infection (**Figure 4A**).

Various other metabolic pathways are differentially altered by *Leishmania* infection, including glutathione. Indeed, the C57BL/6 network module includes glutathione S-transferases, Gsta2, Gsta5, the transcripts of peroxiredoxin (Prdx1) and that of the multidrug resistance protein 1 (MRP1/ABCC1) (**Figure 3B**).

Taken together, these differentially *Leishmania* induced genes, allowed us to postulate a hypothesis involving these various genes and resulting in differences between the two types of macrophages in the production, storage and transport of NO that could explain the resistance of C57BL/6 mice (**Figure 4B**).

## Discussion

In the present work we emphasize the crucial role of macrophage gene expression background in determining its response to L. major infection. Moreover, combining gene expression analysis with network propagation proves to be an effective strategy for identifying dynamically altered mouse strain-specific networks, which contain mechanistic information about these contrasting responses to infection. Comparison of the contrasting response of different mouse strains, reflecting differences in host response, would help define key determinants of an effective response. Our findings showed that the transcriptomic signatures of M-CSF in BALB/c and C57BL/6 BMdMs are significantly different with more than 200 genes differentially expressed between the two mouse strains. The graph-based analysis of the time-resolved gene expression responses upon *Leishmania* infection with network propagation, and using the DEGs in uninfected macrophages as seeds, identified pathways and biological processes that were activated in infected cells and driven by the host differences.

Global transcriptomic events associated with M-CSF-dependent monocyte-to-macrophage differentiation have been analyzed and associated with major changes in the global transcriptomic profile (17). Moreover, differential basal backgrounds in non-infected BALB/c vs. non-infected C57BL/6 L929 differentiated macrophages have also been previously reported (6). Among the DEGs between the two mouse strains, our analysis showed that Cathepsin E (**Supplementary File 1**) is one of the most strongly modulated genes in the BALB/c derived macrophages. Indeed, a polymorphism in the promoter of this gene, disrupting the consensus binding sequence of the PU.1. transcription factor has been previously reported in hematopoietic cells from C57BL/6J mice (18). 25% of the DEGs genes belong to the immune response pathway, and half of the remaining genes are linked to genes related to the same pathway (**Figure 1**). Among the BALB/c DEGs, we found Cxcl14 and Arginase 2 (Arg2). Cxcl14 is involved in antimicrobial immunity preceding the development of the inflammatory response (19) and plays a role in the course of inflammation (20). Indeed, Cxcl14 analogs effectively inhibited the production of nitric oxide, MCP-1, TNF-α and IL-6 from LPS-stimulated RAW264.7 cells (21). Arg2 promotes *H. pylori* immune evasion by restricting M1 macrophage activation and polyamine metabolism (22), macrophage iNOS protein expression and thus NO production, and pro-inflammatory cytokine responses (23). Its expression, together with Cxcl14 in M-CSF-differentiated BALB/c macrophages, might therefore contribute to limit NO production and cytokines synthesis in these cells in response to *Leishmania* infection. Furthermore, in a different system, it has been reported that secreted Cxcl14 promotes the recruitment of alternatively activated M2 macrophages (24). In contrast, in C57BL/6 monocytes, M-CSF differentially induces the two mouse Nlrp1 (NLR family pyrin domain containing 1) paralogues, Nlrp1a and Nlrp1b, that are able to assemble into an inflammasome complex (25, 26). NLRP1b is the most well characterized and has been shown to activate the inflammasome during infection with *bacillus anthracis* (27, 28), *Listeria monocytogenes*, *Shigella flexneri* (29) and *Toxoplasma gondii* infection (30, 31). The ubiquitin system has emerged as fundamental for the control of the inflammatory response and in particular for the activation of the inflammasome (32). Indeed, autoproteolytic processing within the function-to-find domain (FIIND) splits NLRP1b into two fragments that remain non-covalently bound together. The N-terminus is ubiquitinated, releasing the C-terminal fragment. The E3 ubiquitin ligases, which ensure the ubiquitination of the N-terminal part and thus its degradation by the proteasome, are therefore necessary for the activation of NLRP1. Two E3 ubiquitin ligases, Trim12 and ITCH, are differentially expressed in C57BL/6 differentiated macrophages and may play a role in NLRP1 activation.

The immune response plays a central role in determining disease outcome. The differences in the M-CSF induced basal transcriptomic profile could impact the macrophage response to infection. In particular, these profiles could account for the limited amplitude of the inflammatory response observed in *Leishmania*-infected BALB/c BMdMs, while the conditions that prevail in uninfected C57BL/6 BMdMs could explain the greater magnitude of the immune response induced by *Leishmania* infection in resistant BMDMs (**Figure 2**). Similarly, *L. amazoniensis* has also been reported to induce a stronger immune response activation in BMDMs from C57BL/6 mice as compared to BALB/c mice (6). *In vivo*, these different amounts of cytokines/chemokines produced by the macrophages in different backgrounds could affect leukocyte recruitment, especially at the site of infection. As *L. major* proliferation relies on bone marrow-derived cells freshly attracted to the site of infection where they differentiate into macrophages (33), this will affect the proliferation and the spread of the parasite and therefore the disease progresses.

The network propagation approach, using the DEGs in uninfected macrophages as seeds, highlighted pathways and biological processes that depend on the DEG for their activation. The over-represented pathways in each largest module genes led to 65% of pathways that are different between the two mice strains. By analyzing each of these modules, we reveal various genes involved in cellular processes that have already been described as altered during infection as well as novel genes. Indeed, in the C57BL/6 network, we find the macrophage Migrating Inhibitory Factor (MIF) and the endoplasmic reticulum (ER)‐resident protein Stimulator-of-interferon genes (STING). MIF promotes the release of several pro-inflammatory cytokines (TNFα and IL-1), and the activation of inducible NO synthase (iNOS). It also orchestrates the normal flow of leukocytes in inflammatory tissues and is needed for protection against various protozoan infections (34). Indeed, MIF release is induced by *T. cruzi* infection and leads to parasite killing (35) and MIF-deficient *T. brucei*-infected mice showed a decreased inflammatory response and reduced infiltration of pathogenic monocytic cells and neutrophils (36). MIF is protective in *Leishmania* infection. It increases *Leishmania* elimination (37)while Mif−/− mice are highly susceptible to *L. major* (38). Furthermore, Mif polymorphism is associated with cutaneous leishmaniasis outcome (39).

In many different cell types, the cyclic GMP-AMP (cGAMP) synthase (cGAS)-STING signaling axis, results in the phosphorylation and dimerization of the transcription factor IRF3, triggering/eliciting the transcription of hundreds of interferon stimulatory genes (ISGs) and therefore a strong type I interferon response against microbial infections (40). The released DNA might also be from host origin and mtDNA, released by immune cells after LPS stimulation is immunostimulatory and able to contribute to NLRP3 activation through cGAS-STING stimulation (41). Furthermore, different nucleic acid sensors belonging to the Ifi-200/HIN-200 gene family are induced by M-CSF and differentially expressed in BMdMs obtained from the two mice strains. In response to *Leishmania* infection, Ifi proteins, either alone or in cooperation with cGAS-STING, could sense cytosolic DNA and initiate innate immune responses against microbial infection (42). More recently, STING-dependent type I IFN production has been shown to regulate the metabolic changes induced by *Mycobacterium tuberculosis* infection in BMdMs (43). Activation of STING requires palmitoylation once translocated to the Golgi (44) and an ER-associated Zinc Finger, DHHC-Type Palmitoyltransferase 17 (ZDHHC17), is present in the shared network module.

Thus, Mif and Sting expression in the C57BL/6 module is consistent with the ability of these mice to restrict the parasite infection and to induce the repolarization of M2 macrophages to an M1 phenotype (45).

We checked plausibility of the network approach by validation of well-known marker genes and pathways. The inferred network modules, however, carry also many novelties and potential innovative biomarkers and cross-talk between different pathways. To highlight this, we performed additional pathway over-representation analysis with the 194 genes found either in the C57BL/6 or in the BALB/c network modules and found multiple enriched pathways, in particular metabolic and signaling pathways (**Supplementary Table S3.5**; **Supplementary Figure 4A**). A gene prominently expressed in many of these pathways is *Pla2G4a* (phospholipase A2 group IVA) which is higher expressed in BALB/c compared to C57BL/6 macrophages (**Supplementary Figure 4B**). Interestingly, in a recent study *Pla2G4a* among others was found as a signature of infected mouse liver after infection with *L. donovani* (46). In total, 52 genes associated with metabolism are among the 194 genes of the two network modules (**Supplementary Table 3.6**). Another metabolic gene that appears higher responsive in infected BALB/c compared to C57BL/6 macrophages is *Gbe1* (1,4-Alpha-Glucan Branching Enzyme 1). This enzyme is essential for glycogen production and thus energy storage and its upregulation has only recently been identified as a potential biomarker of early infection with *L. donovani* (46).

Cellular metabolism plays a crucial role in the functional phenotype acquired by macrophages and in the regulation of macrophage immune response and inflammation. Metabolic reprogramming has therefore emerged as a promising strategy for fighting microbes. Several genes identified in the C57BL/6 and/or BALB/c network belong to cellular metabolism, highlighting the importance of metabolic pathways in macrophage response to *Leishmania* infection. We and others have previously reported the metabolic reprogramming of infected macrophages toward aerobic glycolysis (47, 48). This change in host energy metabolism is reflected in the transcription upregulation of key glycolytic enzymes and glucose transporters. Macrophage metabolic reprogramming and the induced glycolytic enzymes transcription during infection, is under the control of HIF-1 transcription factor (HIF-1). Although commonly over-represented, the enrichment p-value and HIF-1 signaling target genes are different with respect to the two mouse strains (data not shown). Limiting host glycolysis by targeting metabolic enzymes may be a strategy for *Leishmania* to facilitate its growth (49). Indeed, as assessed by qRT-PCR, transcript levels of these genes are higher in C57BL/6 BMdMs (**Supplementary Figure 3**) which may also contribute to the enhanced immune response observed in these cells. Indeed, the macrophage immune response is also metabolically controlled, and various glycolytic enzymes have been shown to play a role in cytokine expression (50). Conversely, cytokines also regulate metabolic pathway (51). Disruption of TCA cycle contributes to accumulation of citrate that is transported from the mitochondria to cytosol, where it is converted by citrate lyase to acetyl CoA and used for FA synthesis. The multifunctional fatty acid synthase enzyme (FASN), present in the BALB/c module network, catalyzes the condensation of acetyl-CoA and malonyl-CoA and leads to the generation of palmitic acid, a fully saturated 16-carbon FA. Condensation of serine and palmitate (PAL; derived from palmitoyl-CoA) initiates the synthesis of sphingolipids that represent a key class of bioactive lipids, such as sphingosine 1P. S1P affects macrophage biology in different ways (52) and its biological effect is regulated by the relative expression level of the different S1P receptors (S1PR) on immune cells. S1PR2, expressed by innate immune cells, is also present in the BALB/c module network. The role of S1P signaling during *Leishmania donovani* infection has been investigated and has been reported to decrease the expression of S1PR2. This inhibition significantly decreases IL-12 secretion and increases the parasite load as a result of increased IL-10 secretion (53).

Another metabolism highlighted by our analysis is that of arginine, which ultimately leads to the production of a set of biochemically diverse products, including nitric oxide. Network propagation results concerning genes related to this pathway suggest that its regulation is different depending on the genetic origin of the macrophages. Indeed, while Nos2 is present in both BALB/c and C57BL/6 networks, Ass1 is specific to the one of C57BL/6. Arginine can directly be acquired from the extracellular medium but can also be obtained from citrulline recycling *via* Ass1 expression, when extracellular arginine is depleted. This has been demonstrated during mycobacterial infection of murine macrophages (54). Moreover, recycling activity is required for controlling *Mycobacterium bovis* infection, as Ass1-deficient macrophages fail to scavenge citrulline under arginine-depleted conditions, rendering them unable to control mycobacterial infection (54). Citrulline recycling *via* ASS1 could therefore provide the arginine required for the sustained activation of iNOS observed in C57BL/6-derived BMdMs (**Figure 4**) as arginine from citrulline recycling is the preferred substrate for NO production (55). Therefore, despite the enhanced transcription level of both Arg1 and NOS2 observed in C57BL/6-derived BMdMs, citrulline recovery would benefit NO synthesis.

The activation of ASS1 in BMdMs derived from resistant mice could be driven by a decreased uptake of available extracellular arginine by these macrophages. Indeed, previous studies reported reduced expression of the arginine transporter (Slc7a2), in C57BL/6 macrophages (compared to BALB/c) (7) resulting in decreased uptake of available extracellular arginine in response to cytokines (56). Our transcriptomic results show that *Leishmania* infection also induced a more robust expression of Slc7a2 mRNA in BMdMs derived from BALB/c (data not shown). Therefore, the reduced expression of the arginine transporter in C57BL/6 BMdMs would result in a low capacity to import extracellular arginine, inducing activation of Ass1 and the recycling of citrulline that maintain arginine availability and sustain NO production.

We have previously reported that several enzymes of the GSH system are differentially regulated in resistant and susceptible BMdMs suggesting glutathione accumulation in macrophages derived from resistant mice (57). In the C57BL/6 network module, we also notice the presence of the multidrug resistance protein 1 (MRP1/ABCC1) which in addition to its role in drug efflux, contributes at the physiological level to GSH export. Moreover, MRP1 has been reported to be involved in the NO-mediated Fe and GSH efflux (58). Enhanced NO production and GSH accumulation induced by the parasite infection in C57BL/6 mice, could result in NO efflux, in the form of dinitrosyl iron complexes (DNICs), mediated by the glutathione (GSH) transporter, MRP1/ABCC1. Especially that in the C57BL/6 module is also present the transferrin receptor (TFRc), implicated in iron uptake. NO storage and transport system protects the cell from large quantities of endogenously generated NO and might be responsible for delivering large quantities of cytotoxic NO as DNICs *via* MRP1 from M1macrophages to infected cells. Finally, besides its direct antimicrobial activity, NO play a key role in reshaping macrophage metabolism (59) and might also restrict the local enrollment of inflammatory cells and therefore limit the accessibility of cellular niche required for pathogen replication (60).

The genes present in our lists contain also many others whose involvement in the response to *Leishmania* infection has not yet been investigated. These genes that belong to different pathways and biological processes, could be interesting candidates to explore for a better understanding of the macrophage-*Leishmania* interaction.

The finding that the networks stemming from these differentially expressed genes yield a compilation of genes and pathways recognized as crucial in the macrophage reaction to *Leishmania* infection emphasizes the significance of the fundamental transcriptomic profiles of macrophages, and thus, of these DEGs, in driving macrophage polarization and its response to parasite infection. They are therefore, an interesting lead that can yield valuable results such as the identification of potential biomarkers that can improve disease screening and provide drug targets. Further work and *in vivo* validations in mouse models are required to identify and assess these potential biomarkers. Their applicability to humans should be validated in the human cells and models before being used to guide personalized treatment decisions in the clinic.

## Data availability statement

Publicly available datasets were analyzed in this study. This data can be found here: GSE31995 and GSE31996.

## Author contributions

AB, RH, and LG-T conceived this study and were in charge of overall direction and planning. They also analyzed the data and wrote the manuscript. CB, IR, SR, KT, GB, and HT generated and contributed to the interpretation of the results and provided critical feedback. AB, RH, and CB generated the figures. All authors contributed to the article and approved the submitted version.

## Supplementary material

Supplementary Figure 1 Network propagation results. **(A)** Histogram of network modules identified from BALB/c dynamic response profiles. X-axis: module number, Y-axis: size of module. **(B)** Histogram of network modules identified from C57BL/6 dynamic response profiles. X-axis: module number, Y-axis: size of module. **(C)** Largest BALB/c network module consisting of 76 genes. Orange nodes refer to seed nodes, grey color are significantly inferred nodes. **(D)** Largest C57BL/6 network module consisting of 95 genes. Orange nodes refer to seed nodes, grey color are significantly inferred nodes.

Supplementary Figure 2 Joint response network nodes. BALB/c-only nodes (blue), C57BL/6-only nodes (red) and nodes found in both response networks and their number of interactions (Y-axis: Degree) in the joint response network. Significant differences are found between BALB/c-only nodes and nodes from both networks (p = 0.042) as judged by an unpaired Wilcoxon rank-sum test. * p< 0.05

Supplementary Figure 3 Genes involved in glycolysis are differentially regulated by L. major in susceptible and resistant bone marrow-derived macrophages (BMdMs). BALB/c (blue (Parasite (P)) black (Killed P (Kp)) filled triangle) and C57BL/6 (red (P) black (Kp) filled square) BMdMs were infected by L. major promastigotes for different times. RT-PCR different glycolytic genes were performed. The graphs show fold change results expressed as the mean ± SD from three independent experiments. *p<0.05 (two-way ANOVA).

Supplementary Figure 4
**(A)** Pathways and pathway crosstalk enriched by the genes of the BALB/c and C57BL/6 response network modules. Each circle represents an enriched pathway. Size of circle reflects pathway size, color reflects significance of enrichment (from light to dark red), thickness of the edges indicate level of overlapping genes in the connected pathways and color of edge reflects the overlap within the genes from the network modules (from grey to red). **(B)** *Gbe1* and *Pla2G4a* RT-PCR. BALB/c (*blue (Parasite (P)) black (Killed P (Kp)) filled triangle*) and C57BL/6 (*red (P) black (Kp) filled square*) BMdMs were infected by *L. major* promastigotes for different times. RT-PCR different glycolytic genes were performed. The graphs show fold change results expressed as the mean ± SD from three independent experiments. **p*<0.05 (two-way ANOVA).
