# Supplementary figures and images for: Host M-CSF induced gene expression drives changes in susceptible and resistant mice-derived BMdMs upon Leishmania major infection
Source: Front Immunol. 2023 Apr 28;14:1111072. doi: 10.3389/fimmu.2023.1111072 (PMC10175952; doi:10.3389/fimmu.2023.1111072)

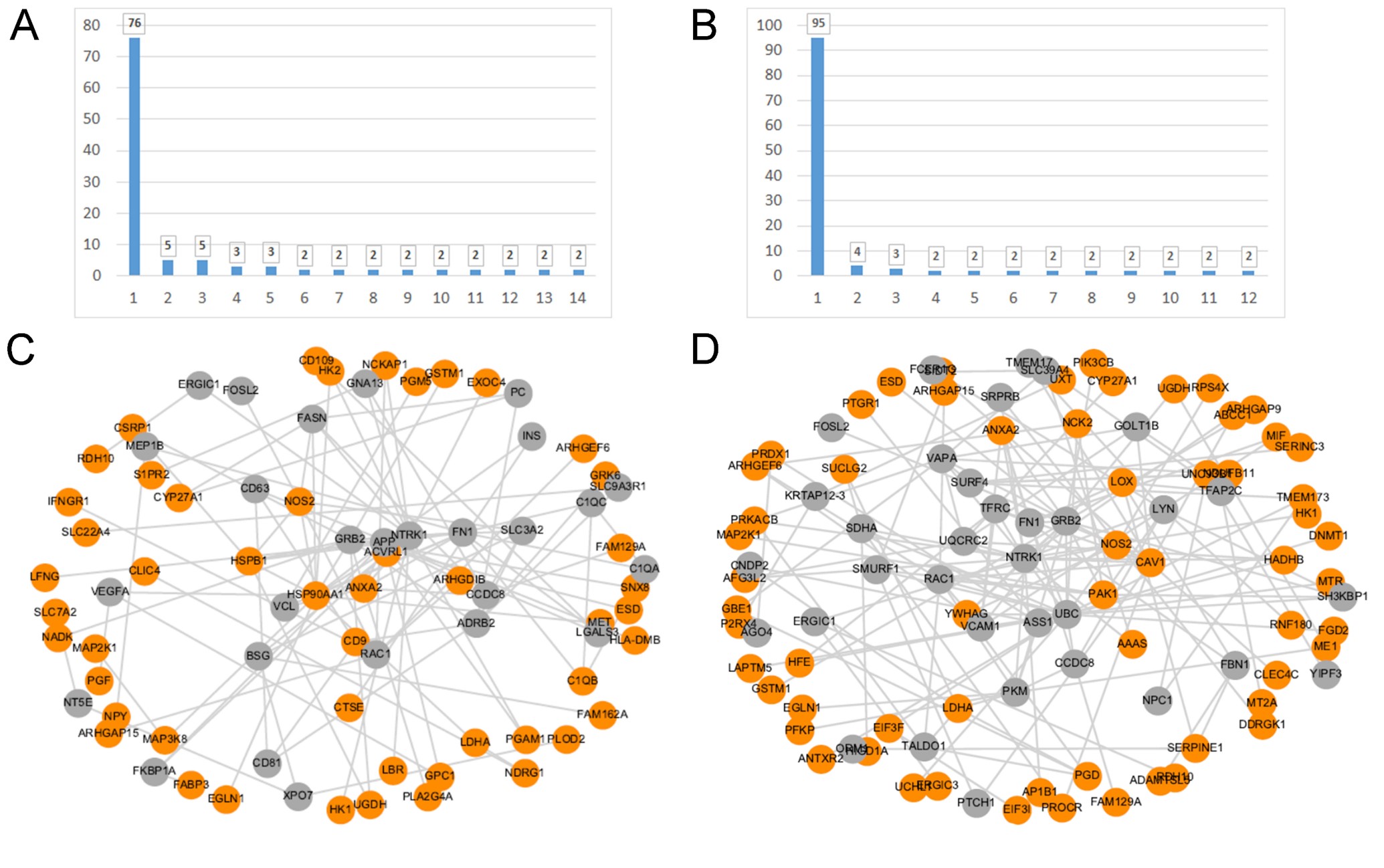

Supplement: Supplementary Figure 1 — Network propagation results. (A) Histogram of network modules identified from BALB/c dynamic response profiles. X-axis: module number, Y-axis: size of module. (B) Histogram of network modules identified from C57BL/6 dynamic response profiles. X-axis: module number, Y-axis: size of module. (C) Largest BALB/c network module consisting of 76 genes. Orange nodes refer to seed nodes, grey color are significantly inferred nodes. (D) Largest C57BL/6 network module consisting of 95 genes. Orange nodes refer to seed nodes, grey color are significantly inferred nodes. [file Image_1.jpeg]

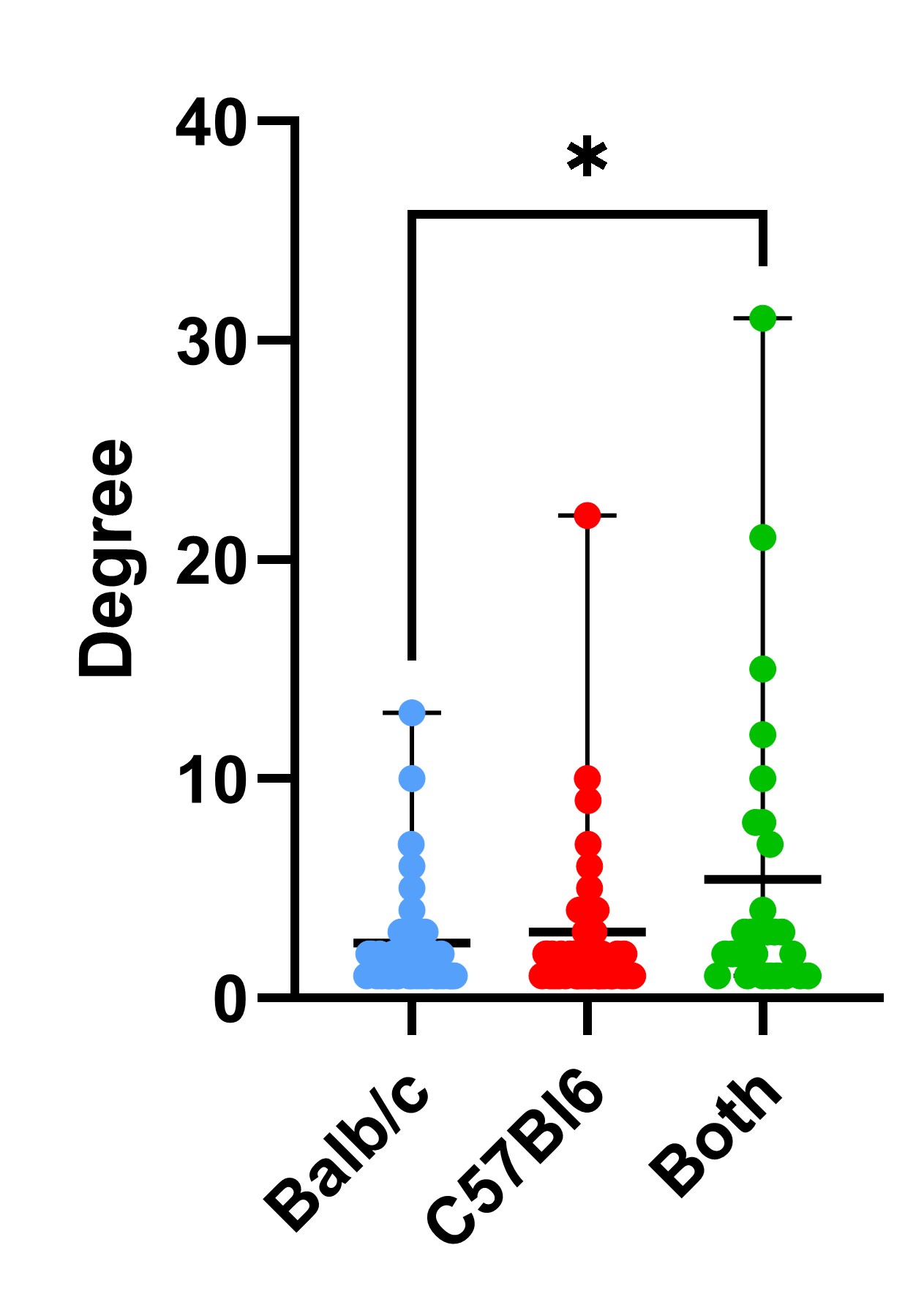

Supplement: Supplementary Figure 2 — Joint response network nodes. BALB/c-only nodes (blue), C57BL/6-only nodes (red) and nodes found in both response networks and their number of interactions (Y-axis: Degree) in the joint response network. Significant differences are found between BALB/c-only nodes and nodes from both networks (p = 0.042) as judged by an unpaired Wilcoxon rank-sum test. * p< 0.05 [file Image_2.jpeg]

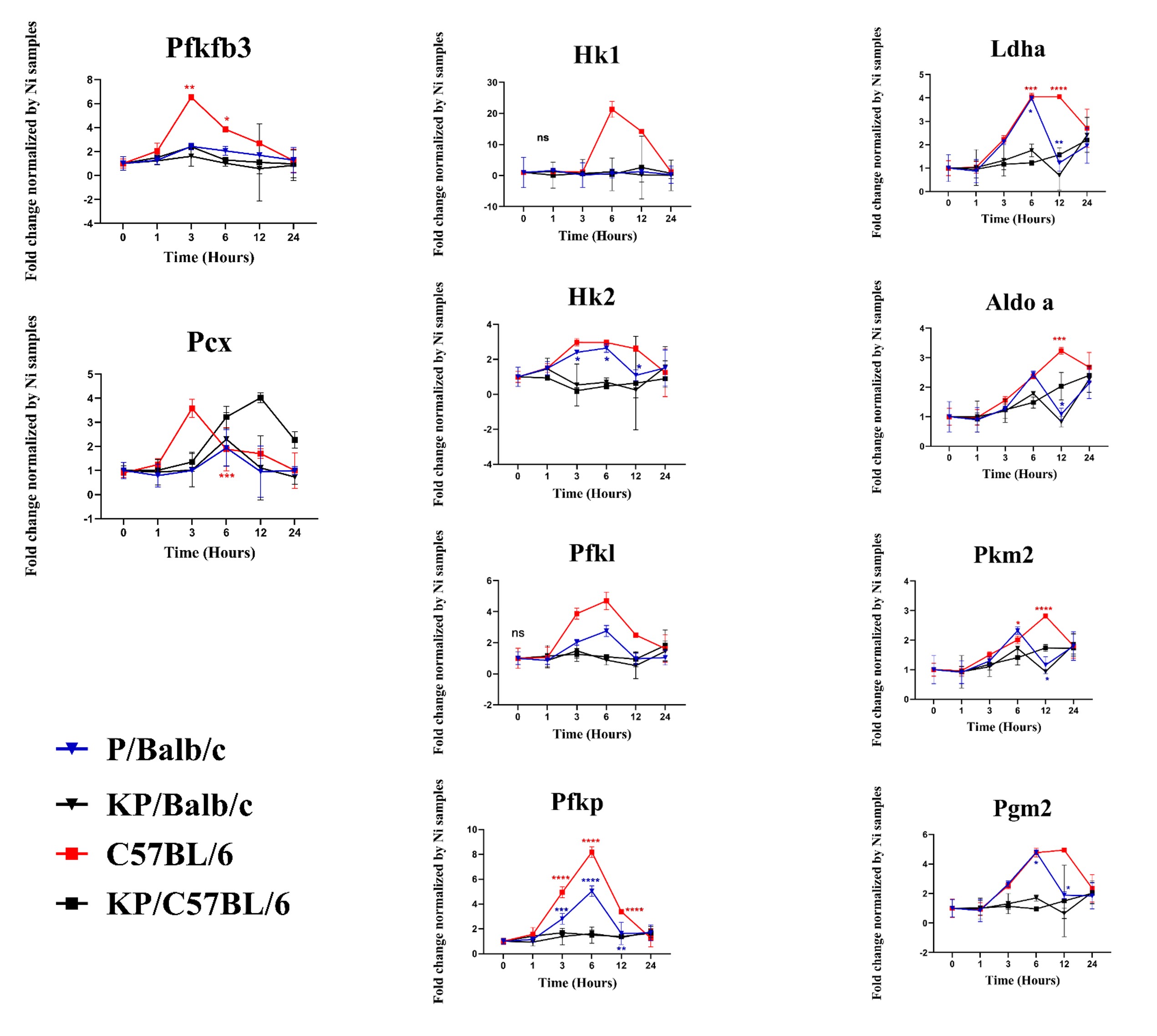

Supplement: Supplementary Figure 3 — Genes involved in glycolysis are differentially regulated by L. major in susceptible and resistant bone marrow-derived macrophages (BMdMs). BALB/c (blue (Parasite (P)) black (Killed P (Kp)) filled triangle) and C57BL/6 (red (P) black (Kp) filled square) BMdMs were infected by L. major promastigotes for different times. RT-PCR different glycolytic genes were performed. The graphs show fold change results expressed as the mean ± SD from three independent experiments. *p<0.05 (two-way ANOVA). [file Image_3.jpeg]

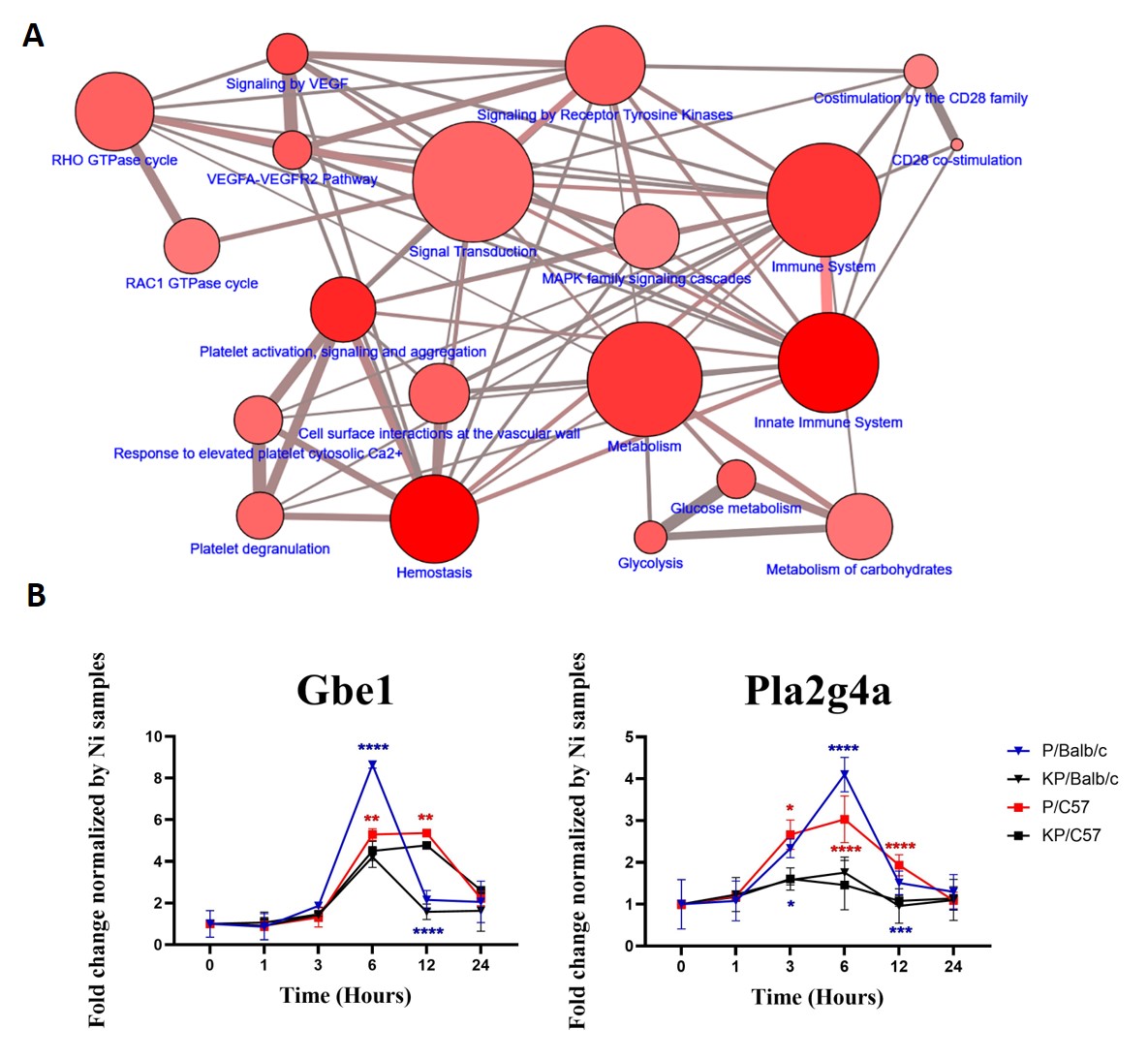

Supplement: Supplementary Figure 4 — (A) Pathways and pathway crosstalk enriched by the genes of the BALB/c and C57BL/6 response network modules. Each circle represents an enriched pathway. Size of circle reflects pathway size, color reflects significance of enrichment (from light to dark red), thickness of the edges indicate level of overlapping genes in the connected pathways and color of edge reflects the overlap within the genes from the network modules (from grey to red). (B) Gbe1 and Pla2G4a RT-PCR. BALB/c (blue (Parasite (P)) black (Killed P (Kp)) filled triangle) and C57BL/6 (red (P) black (Kp) filled square) BMdMs were infected by L. major promastigotes for different times. RT-PCR different glycolytic genes were performed. The graphs show fold change results expressed as the mean ± SD from three independent experiments. *p<0.05 (two-way ANOVA). [file Image_4.jpeg]
